# A novel orally bioavailable compound KPT-9274 inhibits PAK4, and blocks triple negative breast cancer tumor growth

**DOI:** 10.1038/srep42555

**Published:** 2017-02-15

**Authors:** Chetan Rane, William Senapedis, Erkan Baloglu, Yosef Landesman, Marsha Crochiere, Soumyasri Das-Gupta, Audrey Minden

**Affiliations:** 1 Susan Lehman Cullman Laboratory for Cancer Research, Department of Chemical Biology, Ernest Mario School of Pharmacy, Rutgers, The State University of New Jersey, 164 Frelinghuysen Road, Piscataway, NJ, 08854, USA; 2 Karyopharm Therapeutics, Inc., 85 Wells Avenue, Newton, MA, 02459, USA.

## Abstract

Breast cancer is a heterogeneous disease consisting of several subtypes. Among these subtypes, triple negative breast cancer is particularly difficult to treat. This is due to a lack of understanding of the mechanisms behind the disease, and consequently a lack of druggable targets. PAK4 plays critical roles in cell survival, proliferation, and morphology. PAK4 protein levels are high in breast cancer cells and breast tumors, and the gene is often amplified in basal like breast cancers, which are frequently triple negative. PAK4 is also overexpressed in other types of cancer, making it a promising drug target. However, its inhibition is complicated by the fact that PAK4 has both kinase-dependent and -independent functions. Here we investigate a new clinical compound KPT-9274, which has been shown to inhibit PAK4 and NAMPT. We find that KPT-9274 (and its analog, KPT-8752) can reduce the steady state level of PAK4 protein in triple negative breast cancer cells. These compounds also block the growth of the breast cancer cells *in vitro*, and stimulate apoptosis. Most importantly, oral administration of KPT-9274 reduces tumorigenesis in mouse models of human triple negative breast cancer. Our results indicate that KPT-9274 is a novel therapeutic option for triple negative breast cancer therapy.

Breast cancer is a heterogeneous disease and can be classified into at least 5 subtypes: (1) luminal A (usually ER and/or PR+, HER2−, low Ki67), (2) luminal B (usually ER+and/or PR+, HER2+, or HER2− with high Ki67), (3) HER2 positive, (4) basal like, and (5) normal breast like^1^,^2^. These 5 sub-divisions can be even further subdivided. Most basal like tumors are triple negative (lacking ER, PR, and HER2 expression)^3^, and most triple negative cancers have the basal like phenotype. Among the different types of breast cancer, triple negative breast cancer has a particularly poor prognosis. This is due in part to a lack of understanding of the mechanism behind the establishment and maintenance of this type of breast cancer, which consequently limits treatment options. The identification of new biomarkers for the disease is urgently needed to provide effective druggable targets and improve clinical therapy.

KPT-9274 and the closely related KPT-8752 were first identified on the basis of their ability to bind and reduce the steady state level of cellular PAK4 (P21 activated kinase 4) protein, and they were subsequently found to block the activity of NAMPT (nicotinamide phosphoribosyltransferase)^4^. PAK4 inhibition is significant because of the important links that have been found between PAK4 and many types of cancer, including breast cancer. We and others have found PAK4 protein and mRNA levels to be high in a number of breast cancer cells as well as in primary human breast cancer tumor samples^5^,^6^,^7^,^8^,^9^,^10^,^11^. Furthermore, in a study of 80 breast cancer patients with different stages of disease, PAK4 protein levels were shown to increase as the disease progressed, with the highest PAK4 levels being associated with the most advanced stage^8^. In another study of 93 invasive breast carcinoma patients, high PAK4 levels were associated with advanced stage cancer, large tumor size, lymph node metastasis, and poor survival^11^. In another panel of 300 human breast cancers, PAK4 protein was also highly expressed in the more severe grade invasive carcinomas^10^. Our group and others have found that PAK4 protein levels are high in breast cancer cell lines and primary breast cancer tissue^5^,^6^,^7^,^8^,^9^,^10^. In a study of basal like breast cancer, a subset that is frequently triple negative, DNA analysis revealed that the chromosomal region containing the gene for PAK4 was frequently amplified^12^. We found that PAK4 overexpression led to oncogenic transformation in mouse mammary epithelial cells while blocking PAK4 with siRNA inhibited tumor formation of a human breast cancer cell line^6^,^13^. These data suggest a significant role for PAK4 in breast cancer etiology and make it a potential therapeutic target.

The PAK family of protein kinases are important signaling molecules connected to many cellular functions including cell proliferation, migration, and cytoskeletal organization. Aberrant signaling in these pathways are often associated with cancer development and progression^14^. The PAK family consists of 6 members which fall into 2 groups, Group I (PAKs 1, 2, and 3) and Group II (PAKs 4, 5, and 6). Among the group II PAK genes, PAK4 is most frequently linked with cancer^5^,^6^,^7^,^15^,^16^,^17^,^18^,^19^,^20^,^21^,^22^,^23^,^24^,^25^,^26^,^27^,^28^,^29^,^30^. In addition to breast cancer^12^, the PAK4 gene was shown to be amplified in a number of different cancer types, including pancreatic cancer^17^,^24^,^25^, squamous cell carcinomas^26^, esophageal squamous cell carcinoma (ESCC)^31^, endometrioid tumors, ovarian tumors and cell lines^20^, as well as prostate cancer^28^.

Our previous research has shown that when PAK4 is overexpressed in non-transformed immortalized mouse mammary epithelial cells (iMMECs), it results in improper formation of spherical acini in 3D culture. Specifically, elevated PAK4 protein levels lead to increased cell proliferation and survival, decreased apoptosis, filling of the luminal space with cells, increased acinar size, an increase in the outer layer of epithelial cells, and loss of cell polarity^6^. These changes are all hallmarks of precancerous conditions and early stage tumors such as atypical hyperplasias and Ductal Carcinoma *in situ* (DCIS). Even more importantly, the PAK4-expressing iMMECs formed tumors when implanted into the mammary fat pads of mice^6^, providing strong evidence that overexpression of the wild-type PAK4 protein is sufficient to lead to mammary tumorigenesis in mice.

In contrast to its role in carcinogenesis when overexpressed, PAK4 silencing using RNAi in the human breast cancer cell line MDA-MB-231 results in a dramatic reduction in cell proliferation and migration^13^. While cancer cells are generally less susceptible to cell death, PAK4 knockdown dramatically induces apoptosis in these cells. Most strikingly, when these siRNA PAK4 knockdown breast cancer cells are implanted into the mammary fat pads of athymic mice, tumor formation is dramatically disrupted^13^. Additionally, the microRNA, mir-199a.b-3p, which is down-regulated in several types of aggressive cancer, was found to directly target PAK4. mir-199a.b-3p can function as a tumor suppressor and specifically suppresses cell proliferation in breast cancer cells. It also alters the cell cycle while reducing the migratory and invasive activity of breast cancer cells, most likely due to its role in down-regulating PAK4^32^. These data indicate that inhibiting PAK4 can restore many aspects of normal growth in breast cancer cells suggesting a central role for PAK4 in mammary cell transformation.

Because of the link between PAK proteins and cancer^8^,^31^,^33^,^34^,^35^,^36^,^37^, there has been considerable interest in developing PAK inhibitors. KPT-9274, along with the structural analog KPT-8752 (both developed by Karyopharm Therapeutics), function differently from other PAK4 inhibitors in that they reduce the steady state level of PAK4 protein in cells. This reduction is important because PAK4, like other PAK family members, has been found to have several kinase-independent functions^10^,^11^,^38^,^39^,^40^,^41^. For this reason, inhibitors that can reduce PAK4 protein and not just the kinase activity are needed in order to more efficiently block PAK4 in cancer.

In this study we show that KPT-9274 and KPT-8752 are highly effective at blocking the viability of several different breast cancer cell lines, especially three different triple negative cell lines. Most importantly, oral administration of KPT-9274 greatly reduced tumorigenesis in mouse xenograft models of human triple negative breast cancer cell lines. Since KPT-9274 is currently in a phase 1 human clinical trial of patients with advanced solid malignancies (NCT02702492), our data has practical applications to the breast cancer patient population.

## Materials and Methods

### Reagents and Cell culture

KPT-9274 and KPT-8752 from Karyopharm Therapeutics Inc (Newton, MA) were dissolved in dimethyl sulfoxide (DMSO). MCF7, MDA-MB-231 and SkBr-3 cells were maintained in DMEM/F-12 medium supplemented with 10% FBS serum and 1% penicillin/streptomycin. SUM159 cells were maintained in Ham’s F12 medium supplemented with 5% FBS; MDA-MB-468 cells were maintained in RPMI medium supplemented with 10% FBS serum and 1% penicillin/streptomycin. BT-474 were maintained in DMEM medium supplemented with 10% FBS serum, 1% penicillin/streptomycin and 1% glutamine. iMMECs were maintained in Hams F-12 medium supplemented by 10% FBS, 1% penicillin/streptomycin and other supplements. NIH3T3 cells were maintained in DMEM medium supplemented with 10% Bovine Calf Serum, 1% penicillin/streptomycin and 1% glutamine. All cells were maintained at 37 °C and 5% CO_2_.

### Western Blot analysis

Cell lysates (25 μg) were resolved by SDS-PAGE and transferred to PVDF membrane. The membrane was blocked in TBS/T containing 0.1% Tween-20 (TBS/T) and 5% non-fat milk for 1 h. After washing with TBS/T, the membrane was incubated with primary antibody in TBS/T containing 0.1% Tween-20 (TBS/T) and 5% BSA overnight. After washing three times with TBS/T, the membrane was probed with HRP conjugated secondary antibody for 1 h. After washing three times with TBS/T, the part of membrane corresponding in size to the bands of interested protein was excised, and the immunocomplexes were visualized by Luminata Western HRP substrates from Millipore (Billerica, MA). Primary antibodies against PAK4, Cofilin, Phospho-cofilin (Ser3), β-Catenin and Phospho-β-Catenin (Ser675) and β-actin (Rabbit) and HRP-conjugated anti-rabbit antibodies were obtained from Cell Signaling Technologies (Danvers, MA, USA). Primary antibodies were diluted into TBS/T containing 5% bovine serum albumin at 1:1000. Secondary antibody was diluted into TBS/T containing 5% non-fat dry milk at 1:5000. The blots were analyzed either exposing the blots to X-Ray film, or by using the GeneGnome XRQ-NPC bioimaging system from SYNGENE (Cambridge, UK). This system utilizes a software GeneSys (Version 1.5) which automatically selects the right imaging conditions for each blot, backgrounds are adjusted as necessary, and the results are displayed digitally, without the use of X-Ray film. Quantitation of western blots were carried out using image J software. Protein is normalized to β-actin and results are plotted as percent of control, where the band intensity for control is set as 100% for each protein.

### MTT assays

MDA-MB-231, MDA-MB-468, SUM159, MCF7, SkBr-3, BT-474, WT iMMEC and NIH3T3 were seeded into 96-well plates at 2000 cells/well. Cells were treated with KPT-9274 or KPT-8752 from Day 0 to Day 4. At each time point, 10 μl of MTT-I solution (thiazolyl blue tetrazolium bromide, M2128, Sigma-Aldrich, St, Louis, MO) was added into each well and incubated for 5 h, followed by addition of 100 μl of MTT-II solution (distilled water with 10% SDS and 0.01 M HCl). The plate was then incubated overnight and the absorbance was measured with a spectrophotometer (Tecan US, Durham NC) at 560 nm.

### Apoptosis assay

MDA-MB-231, MDA-MB-468, SUM159, MCF7, BT-474, SkBr-3 and WT iMMECs were incubated with 15 μM of DMSO or 3 μM KPT-8752 or 1 μM KPT-9274 for 72 h. Apoptosis was assessed by staining with Annexin V and propidium iodide. Annexin V is a membrane phosphatidylserine (PS) binding protein. It binds to the cells early in apoptosis, which is characterized by PS being flipped to face the outer membrane of the cells. Propidium iodide can enter the cell and bind to nucleic acid, but only after the membrane has begun to rupture, a characteristic of more advanced apoptosis. To assess binding by Annexin V and propidium iodide, cells were trypsinized into single cell suspension, counted, washed with 1X Annexin V binding buffer and stained with Annexin V and Propidium Iodide (BD Pharmingen FITC Annexin V Apoptosis Detection Kit II, BD Biosciences, Franklin Lakes, NJ). The cells (1 × 10^5^) were incubated with Annexin V and Propidium iodide for 15 minutes in the dark at room temperature, then washed with 1X Annexin V binding buffer and analyzed by flow cytometry using a Gallios Cytometer (Applied Biosystems, Foster City, CA).

### Animal Studies

All animals were approved by the Institutional Review Board for the Animal Care and Facilities Committee of Rutgers University. All methods were approved by the guidelines at Rutgers University, and methods were carried out according to the guidelines and regulations of the animal care and facilities committee at Rutgers. Female nude mice (5–6 weeks old, weighing 20–25 grams) were purchased from Charles River Laboratories (Wilmington, MA). They were allowed to acclimatize to the facilities for two weeks following which MDA-MB-231, MDA-MB-468 and SUM159 cells were injected subcutaneously in both the flanks of the mice, at 10^6^ cells per site, in a 100 μl mixture containing Matrigel (BD Biosciences) and Hank’s Buffer (Gibco) at a 1:1 ratio. Seven days post injection, mice were treated with placebo or KPT-9274 (100 mg/kg or 150 mg/kg) orally twice a day/four days per week. Tumor size and total body weights were measured twice weekly. Tumors were measured with a vernier caliper, and tumor volume (V; mm^3^) was calculated using the equation V = D ∗ d^2^/2 where D (mm) and d (mm) are the largest and smallest perpendicular diameters. After sacrificing the animals, tumors were excised, weighed, and snap frozen in liquid nitrogen for western blot analysis.

### Statistical Analyses

Statistical analysis was done using a two-tailed t-test assuming unequal variance with error bars representing SD. *Represents a P value of < 0.001 and is considered significant.

## Results

### PAK4 protein levels are high in multiple breast cancer cell lines

**Figure 1:**
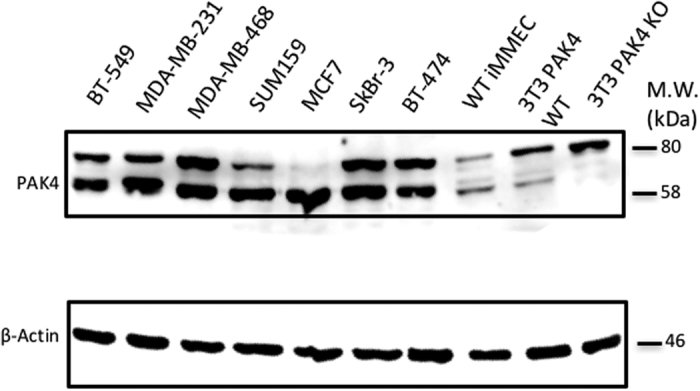
PAK4 is highly expressed in breast cancer cell lines. PAK4 protein levels in seven breast cancer cell lines were assessed by western blot analysis. β-actin was used as a loading control. 3T3 PAK4 WT and 3T3 PAK4 KO are 3T3 cells isolated from wild-type and PAK4 knockout mice, respectively. Knockout cells are used here only for the accurate identification of the PAK4 band. (The membranes were cut prior to exposure so that only the portion of gel containing bands in the size range of PAK4 or β-actin would be visualized, as described in materials and methods).

We assessed the steady state level of PAK4 protein in several breast cancer cell lines by western blot analysis. As observed in Fig. 1, PAK4 levels are high in MDA-MB-468, SUM159, BT-549 and MDA-MB-231 (all triple negative), MCF7 (ER+/PR+), SkBr-3 (HER2+), and BT-474 (PR+/HER2+) breast cancer cell lines. These results are consistent with previous results where the PAK4 levels were high in primary breast cancer tissue^5^,^8^,^10^.

### KPT-8752 and KPT-9274; a novel series of small molecules that can reduce cellular PAK4 protein levels

**Figure 2:**
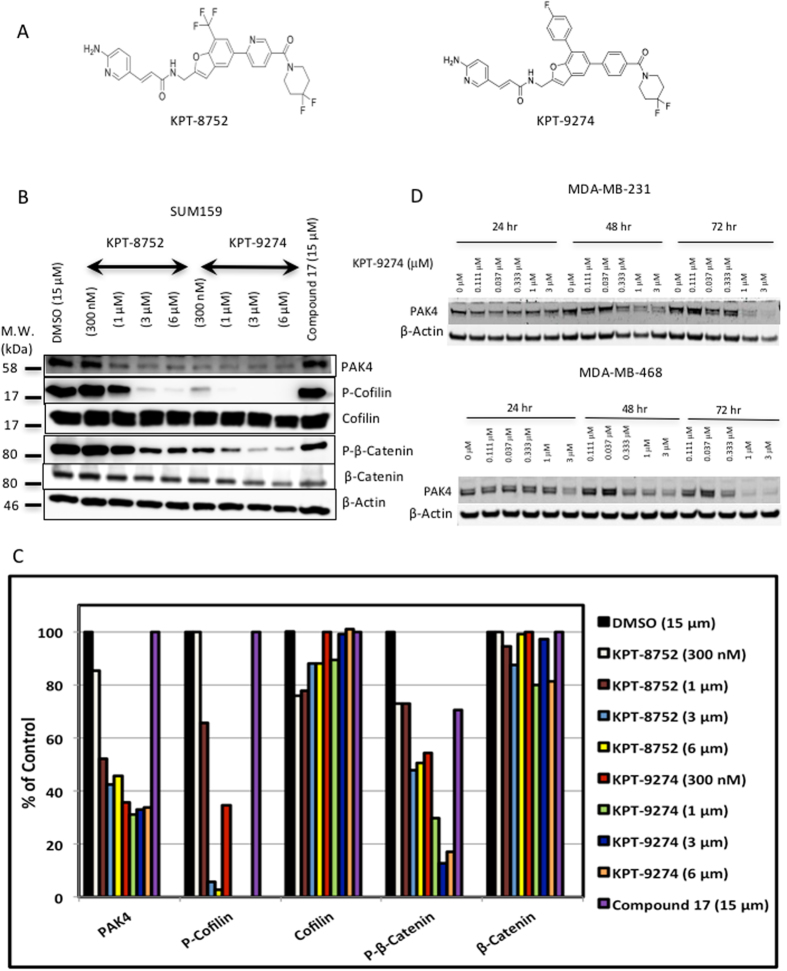
KPT-8752 and KPT-9274 reduce PAK4 protein levels and reduce the phosphorylation of PAK4 downstream targets. (**A**) Structures of KPT-8752 and KPT-9274. (**B**) Western blot analysis of SUM159 cells treated with either KPT-8752, KPT-9274, or compound 17 (72 hr). Western blots were probed with anti PAK4 and anti PAK1 antibodies, and with antibodies against the PAK4 pathway targets β-catenin and Cofilin. β-actin was used as a loading control. (**C**) The intensity of the bands in the western blot in panel B were quantitated using Image J software, and the bands were normalized to the β-actin control. Results are plotted as a percent of control, where the control represents the band intensity for DMSO, and set as 100% for each protein (PAK4, PAK1, Phospho-Cofilin, Cofilin, Phospho-β-Catenin and β-Catenin). This data was from a single experiment, where multiple doses of the inhibitor are represented. (**D**) Western blot analysis of PAK4 levels in MDA-MB-231 cells and MDA-MB-468 cells treated with KPT-9274. (The membranes were cut prior to exposure so that only the portion of gel containing bands in the size range of proteins under investigation would be detected, as described in materials and methods. In the top panel of (**B**) an upper band that represents a non-specific band that reacts with the PAK4 antibody, is spliced out of the figure, in order to focus on the PAK4 band).

KPT-8752 and KPT-9274 (as well as other analogs in the series) were designed and created by Karyopharm Therapeutics. The structures of KPT-8752 and KPT-9274 are shown in Fig. 2A. KPT-8752 or KPT-9274 treatment of SUM159 cells, a triple negative breast cancer cell line, reduced PAK4 protein substantially after 72 h of treatment (see Fig. 2B,C). In contrast, a previously published PAK4 inhibitor, compound 17 42, which blocks PAK4 kinase activity, does not reduce PAK4 protein. In addition to PAK4, we also analyzed the level and phosphorylation status of several PAK4 downstream targets. Serine 675 of β-catenin was shown to be a direct phosphorylation substrate of PAK4^43^. We found that phospho-S675-β-catenin was sharply reduced in response to either KPT-8752 or KPT-9274. The phosphorylation of Cofilin also occurs downstream to PAK4 signaling^44^. We found that KPT-8752 and KPT-9274 treatment of breast cancer cells reduced Phospho-Cofilin as expected. In addition to SUM159 cells, KPT-9274 also reduced PAK4 protein in two other triple negative breast cancer cell lines; MDA-MB-231 and MDA-MB-468 cells, which was most noticeable after 48–72 hours of treatment (Fig. 2D). KPT-8752 had a similar effect in these cells (data not shown).

### KPT-8752 and KPT-9274 block cell growth in several human breast cancer cell lines

**Figure 3:**
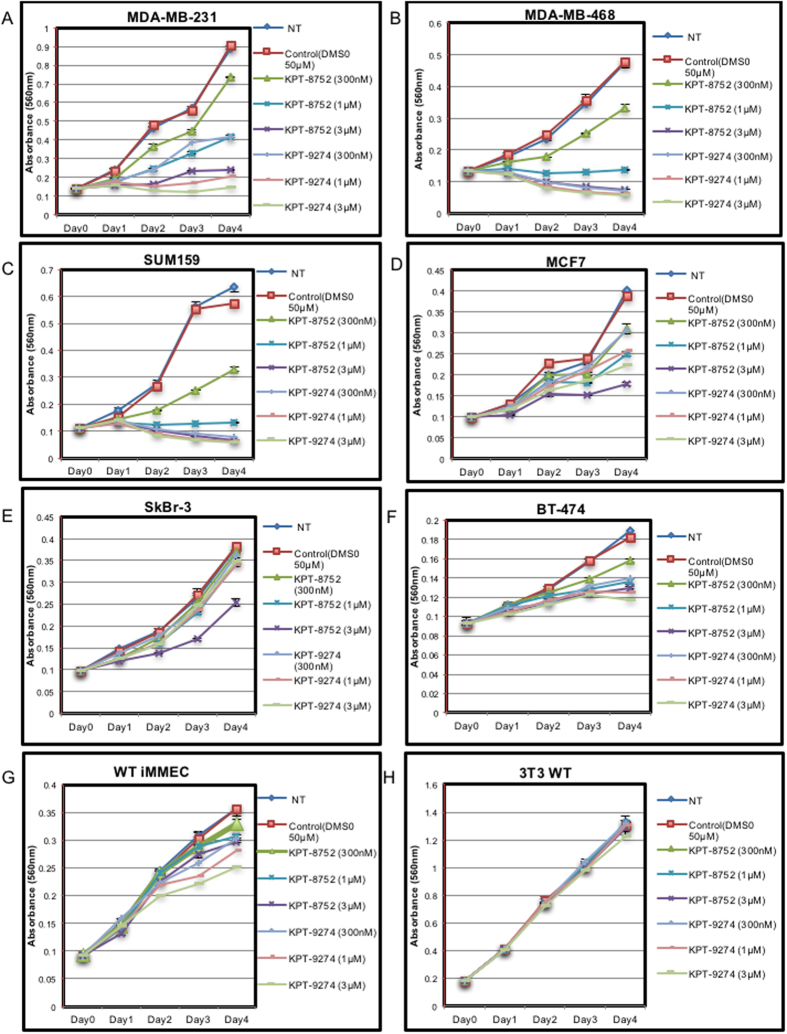
Treatment with KPT-8752 and KPT-9274 leads to a decrease in cell proliferation in several breast cancer cell lines. Cells were plated overnight and treated with DMSO (control), KPT-8752 or KPT-9274 from Day 0 to Day 4. The MTT colorimetric assay was then conducted where the cells were incubated with MTT solutions at different time points. MTT absorbance curves were then analyzed in MDA-MB-231 (**A**) MDA-MB-468 (**B**) SUM159 (**C**) MCF7 (**D**) Sk-Br3 (**E**) BT-474 (**F**) WT iMMEC (control) (**G**) and NIH3T3 (control) cells (**H**). The results, presented as change in absorbance over time, correlate with the number of viable cells over time and can be considered as an indicator of cell proliferation. (Note, the amount of MTT-I absorbed by each cell type varies, and therefore the overall growth rates cannot be compared from one cell type to another). Error bars represent SEM. Data shown is representative of three separate repeat experiments.

The effects of KPT-8752 and KPT-9274 on cell growth was analyzed by using a variation of an MTT cell proliferation assay. The assay is colorimetric and measures the change in the number of metabolically active cells over time as an indicator of cell proliferation (see Fig. 3). Several breast cancer cell lines with high levels of PAK4 protein were plated in tissue culture cluster plates. They were treated with different concentrations of KPT-8752 or KPT-9274 and incubated with MTT solution, and absorbance was measured at different time points as described in materials and methods. After KPT-8752 and KPT-9274 treatment, three of the cell lines, MDA-MB-231 (Fig. 3A), MDA-MB-468 (Fig. 3B), and SUM159 (Fig. 3C), displayed significantly reduced colorimetric change, or viability, over time. For MDA-MB-231 cells, growth was almost completely inhibited in the presence of 3 μm KPT-8752, or as little as 1 μm KPT-9274. For MDA-MB-468 and SUM159 cells proliferation was completely inhibited with 1 μm KPT-8752 or 300 nM KPT-9274. In contrast, the decrease in viability was less pronounced in MCF7 cells (an ER+/PR+cell line, Fig. 3D), and the inhibitory effect was even lower for SkBr-3 cells (HER2+, Fig. 3E) and BT-474 cells (PR+/HER2+, Fig. 3F). Treatment with KPT-8752 or KPT-9274 had no significant effect on cell viability of WT iMMEC or wild-type NIH-3T3 WT cells, but it did inhibit the growth of iMMECs that stably expressed PAK4, suggesting that inhibition of cell proliferation by the compounds is in fact related to PAK4 inhibition (see Supplemental Fig. 1). It is of interest that the triple negative breast cancer cells (MDA-MB-468, MBA-MB-231, and SUM159) were the most responsive to treatment with these compounds, responding to even the lowest concentrations. These results are consistent with previous studies from our lab indicating that PAK4 knockdown with siRNA reduces proliferation of MDA-MB-231 cells^13^.

### KPT-8752 and KPT-9274 induce apoptosis in human breast cancer cell lines

**Figure 4:**
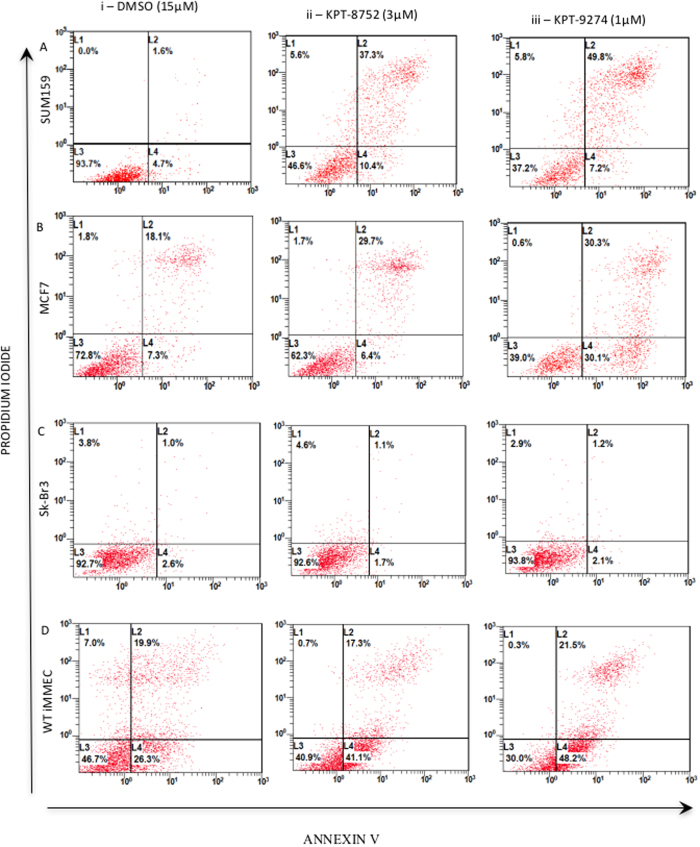
KPT-8752 and KPT-9274 cause an increase in cellular apoptosis in breast cancer cells. SUM159 (triple negative) (**A**), MCF7 (ER+/PR+) (**B**), SkBr-3 (HER2+) (**C**) and WT iMMEC (control) cells (**D**) were treated with either (i) DMSO (15 μM), (ii) KPT-8752 (3 μM), or (iii) KPT-9274 (1 μM) for 72 hours followed by staining for Annexin V/Propidium Iodide (PI). For all cell types, L3 represents the proportion of cells that have low intensity of Annexin V and PI staining and hence have low apoptotic activity, L4 represents the proportion of cells that stain more intensely for Annexin V indicating the early stages of apoptosis, and L2 represents cells that have high levels of Annexin V and PI representing highly apoptotic cells.

Since previous studies indicate that PAK4 siRNA knockdown induces apoptosis in MDA-MB-231 cells^13^, we next studied whether KPT-8752 or KPT-9274 could also affect the survival of additional breast cancer cell lines with various etiologies. The cells were treated with either vehicle control (DMSO), KPT-8752, or KPT-9274 for 72 hours, and apoptosis was measured by staining with Annexin V and propidium iodide (see Fig. 4). The proportion of apoptotic cells corresponds to the number of Annexin V positive and propidium iodide positive cells (L2), and drug induced change in apoptosis is assessed by quantitating the change in L2 in the treated versus untreated cells. SUM159 (Fig. 4A), MDA-MB-231, and MDA-MB-468 cells (Supplemental Fig. 2), (all triple negative), showed a strong increase in apoptosis after treatment with the inhibitors as indicated by high Annexin V and propidium iodide staining (L2). In MCF7 (ER+/PR+; Fig. 4B). although some basal level of apoptosis was seen in the DMSO treated cells, the increase in apoptosis after treatment with KPT-8752 or KPT-9274 was lower than what was observed for the SUM159 cells. For SkBr-3 (HER2+; Fig. 4C) and BT-474 cells (PR+/HER2+; Supplemental Fig. 2), treatment with either compound resulted in almost no increase in apoptosis. WT iMMECs (Fig. 4D) had a basal level of apoptosis, but no increase in apoptosis was observed following KPT-8752 or KPT-9274 treatment. These results are consistent with the MTT assay results demonstrating that the triple negative cells showed the greatest response to inhibitor treatment.

### Oral administration of KPT-9274 blocks tumorigenesis in mouse xenograft studies

**Figure 5:**
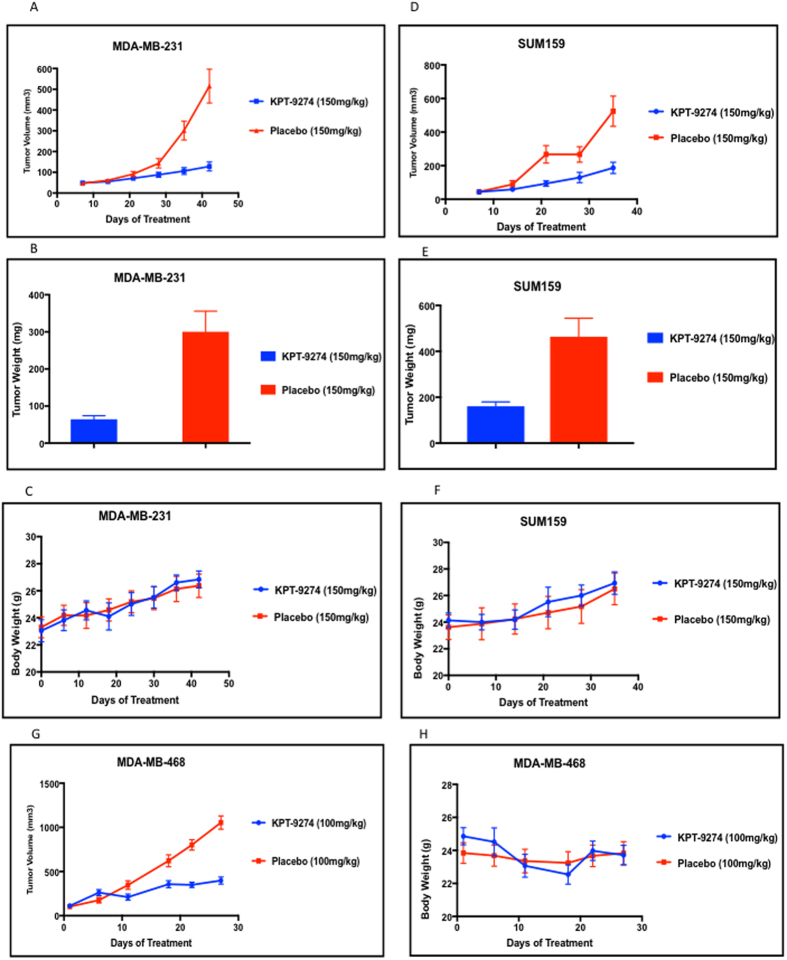
Oral administration of KPT-9274 blocks tumor growth in mice. 10^6^ cells (MDA-MB-231 (**A**,**B**,**C**), SUM159 (**D**,**E**,**F**), or MDA-MB-468 (**G**,**H**), all of which are triple negative breast cancer cell lines, were injected into both flanks of female nude mice. (MDA-MB-231: n = 8, treatment group; n = 10, control group; MDA-MB-468: n = 8, treatment group; n = 7, control group; SUM159: n = 5 treatment group; n = 5, control group). Seven days following injection, mice were treated with orally administered KPT-9274 or Placebo (150 mg/kg PO bidx4 for the MDA-MB-231 and SUM159 cells, or 100 mg/kg PO bidx4 for the MDA-MB-468 cells). Tumor volume (V; mm^3^) was calculated for each cell line (see **A**,**D**, and **G**). Tumor weight was assessed for the MDA-MB-231 cells and the SUM159 cells (see **B** and **E**). Body weight of mice (see **C**,**F**,**H**) was monitored throughout the course of dose administration.

The triple negative breast cancer cells MDA-MB-231, SUM159 and MDA-MB-468 were the most responsive to treatment with KPT-9274 *in vitro* and represent a subtype of breast cancer that is in particular need of novel therapies. We therefore used these cell lines as a model to test the *in vivo* efficacy of the orally bioavailable clinical candidate KPT-9274 (Fig. 5). MDA-MB-231, SUM159 and MDA-MB-468 cells were injected into the flanks of female nude mice. Seven days following tumor cell injection (when tumors were approximately 50–100 mm^3^) mice were administered KPT-9274 or placebo. Treatment continued twice per day for four days per week. Treatment with orally administered KPT-9274 resulted in a significant reduction in the tumor volumes in all three models of the treatment groups as compared to the control groups, and in the tumor weights, which were measured for MDA-MB-231 and SUM159 (see Fig. 5 and Table 1). Treatment did not significantly affect the body weights of the mice (Fig. 5C,F and H).

**Figure 6:**
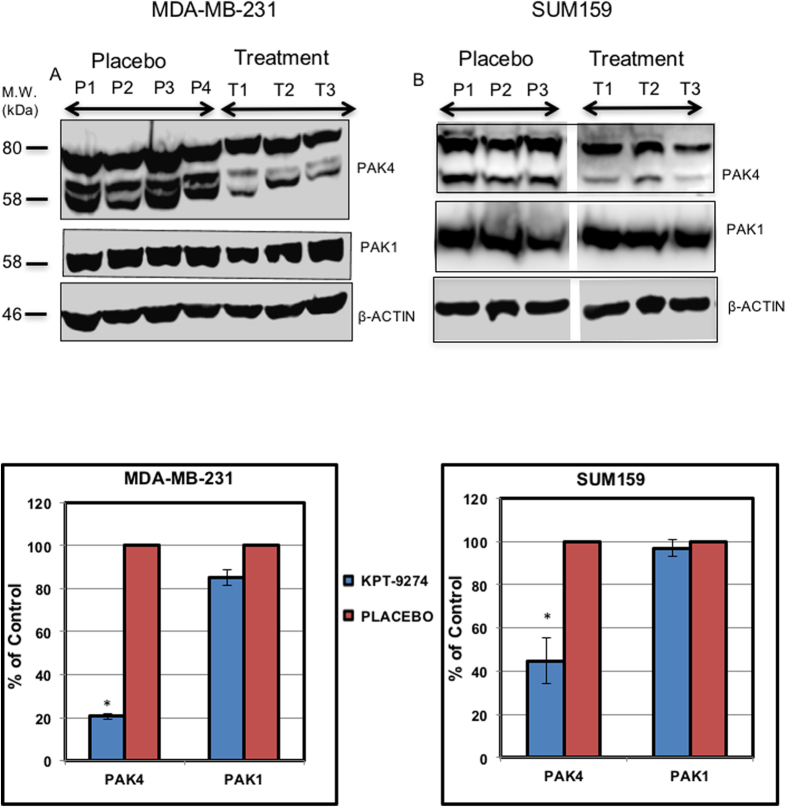
Orally administered KPT-9274 reduces PAK4 protein levels. As the treatment concluded, MDA-MB-231 and SUM159 xenograft tumors were excised and analyzed by Western Blot (**A**). For the MDA-MB-231 mice, tumors from three independent mice that were treated with the KPT-9274 (T1, T2, T3), and from four independent mice that were treated with placebo (P1, P2, P3, P4) were analyzed by western blot, while for the SUM159 cells, tumors from three independent mice treated with KPT-9274 (T1, T2, T3) and three independent mice treated with placebo (P1, P2, P3) were analyzed by western blot. Blots were probed with anti PAK4, anti PAK1, or anti β-actin antibody as a loading control. The intensity of the bands in the blot in panel A were quantitated using Image J software, and the bands were normalized to the β-actin control (**B**). Results are plotted as a percent of control, where the control represents the band intensity for placebo, and set as 100% for each protein (PAK4 or PAK1). Data shown is representative of three separate repeat experiments. (The membranes were cut prior to exposure so that only the portion of gel containing bands in the size range of PAK4, PAK1, or β-actin would be visualized, as described in materials and methods. The top band in the PAK4 panel is a nonspecific band that appears in response to our PAK4 antibody, regardless of the presence of PAK4).

After treatment concluded, the MDA-MB-231 and SUM159 tumors were excised and immunoblotting was performed to measure PAK4 protein levels (Fig. 6). We observed a significant decrease in PAK4 levels in excised tumors from the treatment group, when compared to those from the control (placebo) group. Compared to PAK4, the levels of an off-target protein, PAK1, were not significantly changed after treatment (Fig. 6). Thus, our results indicate that orally administered KPT-9274 reduces the steady state level of PAK4 protein and is capable of reducing growth of the triple negative breast cancer cells MDA-MB-231, MDA-MB-468 and SUM159. These results are also consistent with previous studies indicating that PAK4 knockdown with siRNA blocks tumorigenesis caused by MDA-MB-231 cells^13^.

## Discussion

In this study, we have found that KPT-8752 and KPT-9274 reduce cell proliferation and survival in several triple negative breast cancer cell lines *in vitro*. The most exciting outcome from this study is that KPT-9274 can inhibit breast cancer tumorigenicity *in vivo*, in three independent mouse xenograft models using human triple negative breast cancer cell lines. We examined several subtypes of breast cancer cell lines in the current study and were encouraged to find that treatment with these inhibitors blocks the growth of three triple negative breast cancer cell lines. Because this subtype is generally less responsive to many of the current therapies the data presented here provides a path forward to address the urgent need for novel treatment options for triple negative breast cancer patients.

KPT-8752 and KPT-9274 were identified as small molecules that bind to and reduce the steady state level of PAK4 protein in cells, and they were subsequently shown to inhibit the activity of NAMPT^4^. PAK4 has been found to be elevated in both breast cancer cells and primary breast tumors^5^,^6^,^7^,^8^,^9^,^10^. Furthermore, in basal like breast cancer (a type that is usually triple negative) the chromosomal region containing the PAK4 gene is frequently amplified^12^. However, not all of the cancer cell lines described here were responsive to KPT-8752 or KPT-9274 despite their having high levels of PAK4. Specifically, in contrast to the triple negative cells, treatment with the compounds had only moderate effects on MCF7 cells (ER+/PR+), BT-474 (PR+/HER2+) and SkBr-3 cells (HER2+) *in vitro*, even though all of these cell lines have high levels of PAK4. Investigation of additional breast cancer cells will help to determine whether the different responses we observed were in fact related to the backgrounds of the different cell types, and whether or not they are related to PAK4 status. From a patient population perspective, it will be vital to understand the reason why KPT-9274 was unable to reduce the viability of HER2+ and ER+ cells. One possibility is that KPT-9274 may operate through an additional target(s), in addition to, or possibly even instead of, PAK4, which may play a critical role in the biology of the triple negative cells. Alternatively, the results may indicate that HER2 and ER may promote tumorigenesis by mechanisms that are independent of PAK4. If this is the case, the use of KPT-9274 in combination with HER2 inhibitors or estrogen blockers may be warranted.

PAK4 has several cellular functions that are frequently linked to cancer such as promoting cell survival and proliferation^38^,^39^,^45^, prolonging activation of the ERK/MAP Kinase pathway^6^, and regulating cytoskeletal changes^46^. Because of this link, there has been considerable interest in generating PAK inhibitors. One of the first PAK4 inhibitors identified was PF-3758309, which was broadly active against both group I and group II PAKs as well as several other kinases^47^,^48^. This compound inhibited the growth of a number of tumor cell lines both *in vitro* and *in vivo*^48^,^49^. However, human clinical trials were terminated due to undesirable PK characteristics of the drug (i.e. low bioavailability), adverse side effects, and consequent lack of tumor responses^50^,^51^. A second PAK4 kinase inhibitor, LCH-7749944 52,53, reduced proliferation and invasion of gastric cancer cells *in vitro*, and reduced filopodia formation and cell elongation, but it has not been tested *in vivo*. A third inhibitor, Compound 17 (also called GNE-2861), is potent against Group II PAKs. It reduced the viability and motility of breast cancer cell lines^42^, and it enhanced tamoxifen sensitivity in MCF7 cells^9^, but has poor oral bioavailability^54^. GL-1196 is another small molecule that inhibits PAK4 kinase activity, and suppresses the invasive capability of gastric cancer cells^55^. While clinical studies involving several of the compounds described above have been terminated due to poor bioavailability, improved second generation derivatives of some of these compounds may hold more promise. KPT-9274 is a unique type of inhibitor in that it reduces the steady state level of the PAK4 protein, although the exact mechanism by which it reduces PAK4 levels is not completely understood. Reduction in PAK4 protein is important because like other PAK family members, PAK4 has been shown to have certain functions that are independent of its kinase activity^10^,^38^,^39^,^40^,^41^,^45^. Therefore, reduction of PAK4 protein advantageously reduces or inhibits any oncogenic process that requires the presence of the protein.

While our results indicate that KPT-9274 is a promising agent for triple negative breast cancer treatment, new data is emerging that it may also be effective against other types of cancers^4^,^31^. As a result, KPT-9274 is currently under phase I clinical trials to evaluate its safety, tolerability, and efficacy (NCT02702492), in patients with solid tumors and lymphomas. Among the other available PAK4 inhibitors, we have found that compound 17^42^ can also block the growth of breast cancer cell lines in our system, however it was less effective than KPT-9274 (Rane and Minden, unpublished results). In the future, it would be important to determine whether inhibiting PAK4 simultaneously with different types of inhibitors, could result in even stronger inhibition of PAK4 and inhibition of cancer cell growth. In addition to PAK4, other PAK family members such as PAK1 and PAK2 are often linked to breast cancer^53^^–58^. Therefore, in future studies it will be interesting to determine whether combinations of inhibitors against the different PAK isoforms may be even more effective, particularly in cells that are unresponsive to single isoform inhibition.

Although KPT-9274 and its analogs inhibit PAK4, other PAK isoforms could still be affected. In this study, we found that the reduction in PAK4 levels by KPT-9274 is significantly stronger than the reduction in PAK1, suggesting specificity for the group II PAKs. However, we have not tested the effects of KPT-9274 on the other group II PAK family members, PAK5 and PAK6, in breast cancer cells. Since PAK5 and PAK6 proteins are less frequently associated with breast cancer, our focus for this study was PAK4, but further investigation is warranted.

It is important to consider that in addition to PAK4, KPT-9274 and KPT-8752 also reduces the synthesis of NAD (nicotinamide adenine dinucleotide), by blocking the activity of the enzyme NAMPT (nicotinamide phosphoribosyltransferase)^4^. NAD is involved in a wide range of cellular processes, including DNA repair, and cell signaling, which are also thought to be important in cancer^56^. A direct link between PAK4 inhibition and NAD has not been established, but NAMPT has been reported to activate Cdc42, a known activator of PAK4, during cytoskeletal organization^57^. It is important to consider that many of the effects that we have seen in response to KPT-9274 could also be attributed to NAMPT inhibition, and more work will be required to determine which effects of the compound can be attributed to PAK4 and which can be attributed to NAMPT or possibly even other targets. It should be noted, however, that in breast cancer cell lines, we have seen that KPT-8752 and KPT-9274 do block NAMPT activity, but that inhibition of NAMPT did not correlate with the ability of the compounds to block cell proliferation (Minden lab, unpublished results). While it is important to consider the possibility that KPT-9274 has pleotropic effects and impacts multiple signaling pathways, it is interesting to note that nearly all of the effects observed with the inhibitor are consistent with what has been reported with PAK4 knockdown via siRNA. In particular, our previous work indicated that siRNA knockdown of PAK4 in the triple negative breast cancer cell line MBA-MB-231 reversed many aspects of tumorigenesis. The effects included inhibition of cell proliferation, increased apoptosis, and most importantly, decreased tumorigenesis in mice^13^. These studies strongly support the idea that blocking PAK4 correlates with inhibition of tumorigenesis in triple negative breast cancer. Although more work will be required to determine the exact mechanism by which KPT-9274 operates, our results provide support for the use of this promising clinical candidate in triple negative breast cancer, a disease that is refractory to many of the treatment options currently available.

## Additional Information

**How to cite this article**: Rane, C. *et al*. A novel orally bioavailable compound KPT-9274 inhibits PAK4, and blocks triple negative breast cancer tumor growth. *Sci. Rep.* **7**, 42555; doi: 10.1038/srep42555 (2017).

**Publisher's note:** Springer Nature remains neutral with regard to jurisdictional claims in published maps and institutional affiliations.

## Supplementary Material

Supplementary Figures

## Figures and Tables

**Table 1:** Summary of *In-vivo* data from Fig. 5.

Cell line	No. of tumor (n)		Dosing Regimen	Average Tumor Volume (mm^3^) (Endpoint)		Average Tumor Weight (mg)	
	KPT-9274	Placebo		KPT-9274	Placebo	KPT-9274	Placebo
MDA-MB-231	16	20	150 mg/kg PO bidx4	128 + 21.7	515.1 + 81.6	64.2 + 9.6	300.2 + 55.8
MDA-MB-468	8	7	100 mg/kg PO bidx4	398.7 + 41	1053.8 + 76	—	—
SUM159	10	10	150 mg/kg PO bidx4	187 + 33.7	524.2 + 90.2	160.8 + 19	463.1 + 81.1
