# Supplementary material for: A novel orally bioavailable compound KPT-9274 inhibits PAK4, and blocks triple negative breast cancer tumor growth
Source: Sci Rep. 2017 Feb 15;7:42555. doi: 10.1038/srep42555 (PMC5309789; doi:10.1038/srep42555)
Supplement: Supplementary Figures [file srep42555-s1.pdf]

A novel orally bioavailable compound KPT-9274 inhibits PAK4, and blocks triple negative breast cancer tumor growth

Chetan Rane, William Senapedis, Erkan Baloglu, Yosef Landesman, Marsha Crochiere, Soumyasri Das-Gupta, Audrey Minden

Supplementary figures

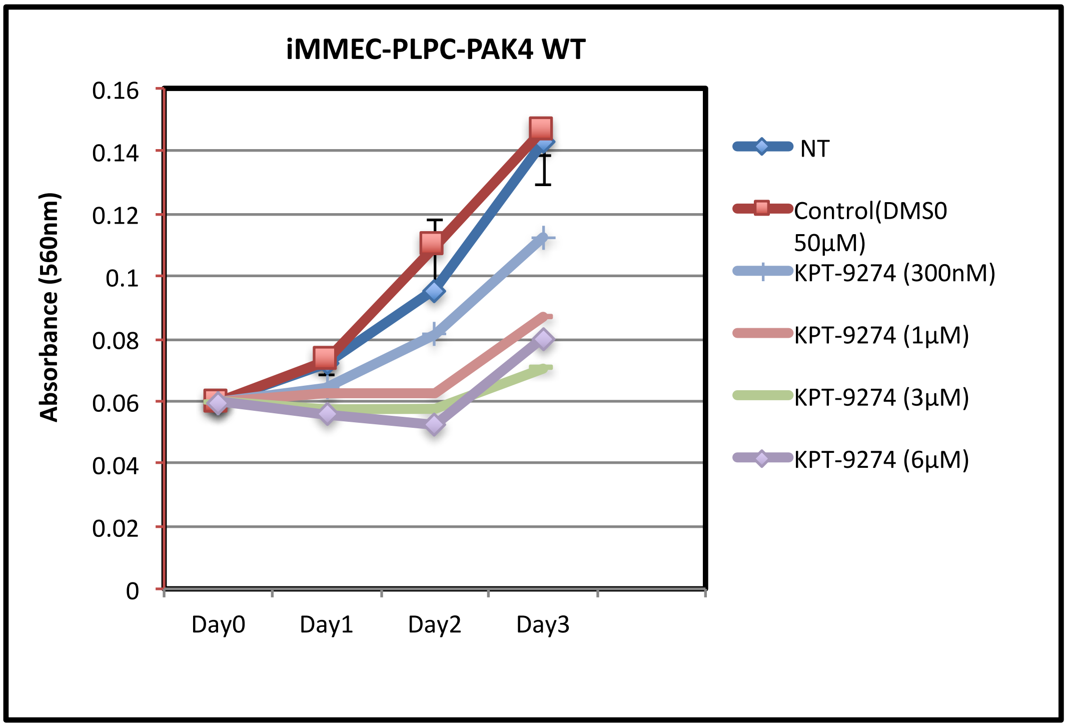

Supplementary Figure 1: iMMECs stably expressing PAK4 were treated with DMSO or KPT-9274, from Day 0 to Day 3. Cells were then incubated with MTT solutions at different time points, followed by measuring absorbance at 560 nm.

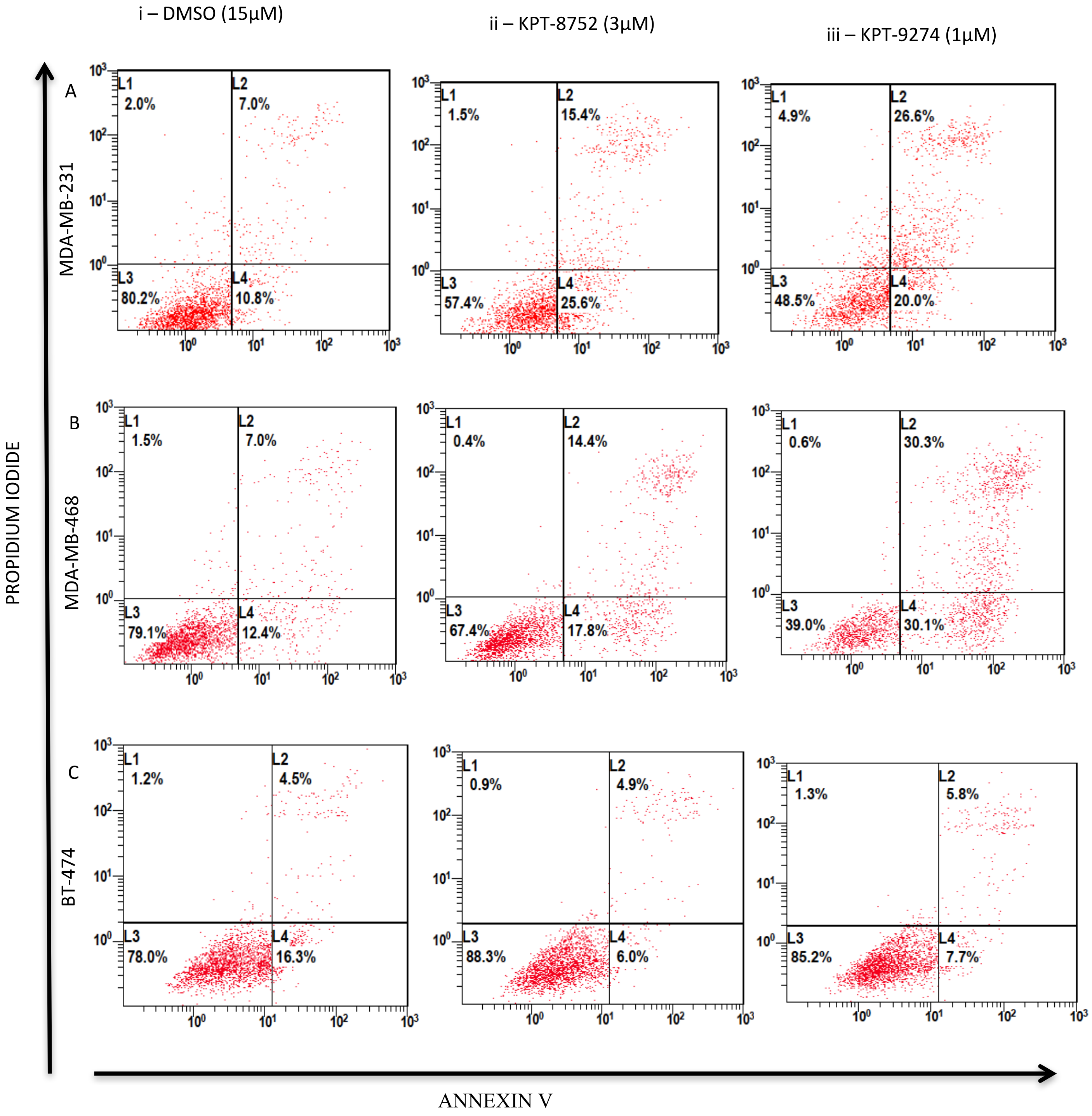

Supplemental Figure 2: MDA-MB-231 (S1A), MDA-MB-468 (Triple Negative) (S1B) and BT-474 (PR+/HER2+) (S1C) were treated with (i) DMSO (15 µM), or (ii) KPT-8752 (3 µM), or (iii) KPT-9274 (1 µM), for 3 days, following staining for Annexin V / Propidium Iodide (PI).
